# Bone transport nails for reconstruction of lower limb diaphyseal defects in patients with bone sarcomas: A case series

**DOI:** 10.1007/s00508-025-02527-5

**Published:** 2025-04-04

**Authors:** Maria Anna Smolle, Silvia Zötsch, Dimosthenis Andreou, Marisa Valentini, Andreas Leithner, Philipp Lanz

**Affiliations:** 1 Department of Orthopaedics and Trauma, Medical University of Graz, Auenbruggerplatz 5, 8036 Graz, Austria; 2 Institute for Interdisciplinary Sarcoma Research and Treatment, Department of Orthopaedic Oncology and Sarcoma Surgery, University Hospital Essen, Essen, Germany

**Keywords:** Bone defect reconstruction, Biological reconstruction, Sarcoma, Bone transport solution, Malignancy

## Abstract

**Purpose:**

Bone transport nails (BTN) are increasingly being used for defect reconstruction in orthopedic surgery, including orthopedic oncology. Herein, we report on preliminary outcomes in three adult sarcoma patients undergoing bone defect reconstruction of the lower limbs with a BTN.

**Methods:**

In this study three patients were retrospectively included; ID1 male, 18 years, Ewing’s sarcoma of the right tibial diaphysis; ID2 female, 30 years, G2 periosteal osteosarcoma of the left femoral diaphysis; ID3 female, 28 years, epithelioid malignant peripheral nerve sheath tumor (MPNST) originating from the right proximal tibial metaphysis. All patients had been treated at a university hospital for primary sarcomas of the lower limbs and underwent defect reconstruction with a BTN (Precice® System, NuVasive GmbH, Globus Medical, Audubon, PA, USA).

**Results:**

Bone defect lengths were 8.5 cm, 14.1 cm and 14.4 cm, respectively. Bone transport time amounted to 5.3 months, 9.1 months and 10.3 months, and time to bony consolidation to 9.1 months, 12.3 months and 14.7 months, respectively (in ID1 and ID3 partially). Patient ID1 required revision surgery for a wire breakage (used for two-level transport) and one screw avulsion. Patient ID2 developed a peri-implant infection that was successfully treated with prolonged antibiotics. In ID3, an occult intraoperative distal femoral fracture was initially treated conservatively. A consecutive varus/flexion deformity and residual ipsilateral limb length discrepancy was addressed via distal femoral osteotomy and retrograde femoral growing nail implantation.

**Conclusion:**

The Precice® BTN can be used for reconstruction of diaphyseal long bone defects in patients with primary malignant tumors; however, candidate patients have to be thoroughly counselled regarding the prolonged immobilization and partial weight-bearing period associated with the lengthening procedure as well as risk for complications and revision surgery.

## Introduction

Reconstruction techniques for diaphyseal or metaphyseal bone defects include massive allografts (with or without fibular autografts), (intercalary) megaprostheses, growing (mega)prostheses (e.g. BioXpand, MUTARS, Implantcast, Buxtehude, Germany), recycled autografts, vascularized autografts, osteoinductive membrane techniques, epiphyseal distraction methods (Canadell technique) [1] and bone transport solutions (e.g. plate-assisted bone segment transport, PABST, Ilizarov circular external fixator) [1–7]. Each of these techniques has specific advantages and disadvantages. For (intercalary) prostheses, one major disadvantage constitutes the foreign material in place, possibly leading to mechanical failure and/or periprosthetic infections, with the prevalence steadily increasing over time [8].

Bone transport nails (BTN) constitute another option to treat diaphyseal bone defects [9, 10], and enable internal distraction osteogenesis similar to external distraction osteogenesis using an external fixator [11]. As an advantage over prosthetic replacement, no to minimal foreign material is left in place after transport has been finished.

A limited number of studies have been published so far on the use of a BTN to reconstruct bone defects following resection of sarcomas [12, 13]. Herein, we report the use of a BTN for reconstruction of large diaphyseal defects in three patients with primary bone sarcomas of the lower limbs.

## Patients, material and methods

In this report three patients (male, 18 years, Ewing’s sarcoma right tibial diaphysis; female, 30 years, G2 periosteal osteosarcoma left femoral diaphysis; female, 28 years, malignant peripheral nerve sheath tumour, MPNST, originating from right proximal tibial metaphysis; Table 1) consecutively treated at a single university hospital for primary diaphyseal bone sarcomas of the lower limbs, who underwent defect reconstruction (defect length 8.5–14.4 mm) with a BTN (Precice® System, NuVasive GmbH, Globus Medical, Audubon, PA, USA) between April 2023 and December 2023 were retrospectively included. The patients were followed until February 2025. The described BTN has been in use for several years at this institution, mainly for bone defects following trauma and infection.

**Table 1:** Detailed description of patient cases

Case (sex, age at diagnosis)	Diagnosis	Oncological treatment	Defect length	Reconstruction details	Transport time	Complications
1 (male, 18 years)	Ewing’s sarcoma, right tibial diaphysis	NCTX (VDC/IE, 9 cycles)	14.1 cm	Nail: BTN Nuvasive 11.5/360 mm tibial nail	10.3 months	1) wire breakage
		ACTX (VDC/IE, 5 cycles)		Osteotomy: two-level tibia		2) screw avulsion
				transport: antegrade + retrograde transport via pulley mechanism		
2 (female, 30 years)	Periosteal osteosarcoma G2 left femoral diaphysis	None	14.4 cm	Nail: BTN Nuvasive 11.5/360 mm femoral nail	9.1 months	1) peri-implant infection
				Osteotomy: distal femur		
				Transport: retrograde		
3 (female, 28 years)	Primary osseous epithelioid MPNST right proximal tibial metaphysis	ACTX (API-AI)	8.5 cm	Nail: BTN Nuvasive 10/300 mm tibial nail + plate (PABST)	5.3 months	1) intraoperative occult distal femoral fracture
				Osteotomy: distal tibia		2) secondary varus/flexion deformity distal femur
				Transport: retrograde		

*ACTX* adjuvant chemotherapy, *API* doxorubicin-ifosfamide-cisplatin, *AI* doxorubicin-ifosfamide, *BTN* bone transport nail, *MPNST* malignant peripheral nerve sheath tumor, *NCTX* neoadjuvant chemotherapy, *PABST* plate-assisted bone segment transport, *VDC/IE* vincristine-doxorubicin-cyclophosphamide ifosfamide-etoposide

All patients received a temporary spacer during sarcoma surgery. In a second surgery either scheduled after completion of adjuvant chemotherapy (CTX; patients 1 and 3) or confirmation of clear surgical margins (patient 2), the BTN was implanted. Patients were followed up on a regular basis with local imaging (X-ray, sonography), as well as computed tomography (CT) scans of the thorax for sarcoma surveillance. During transport with the BTN, no local magnetic resonance imaging (MRI) was carried out in order not to break the nail’s growing mechanism. In all patients bone transport started between the 8th and 10th postoperative day, with 0.75–1 mm transport length per day. Patients 1 and 3 received oncological treatment according to the diagnosed malignancy. Follow-up was scheduled individually based on postoperative healing, bone transport progress and subsequent healing. All patients underwent CT scans to assess bony consolidation during follow-up.

The study was approved by the local institutional review board (IRB-number: 1298/224).

## Results

### Patient 1

**Fig. 1:**
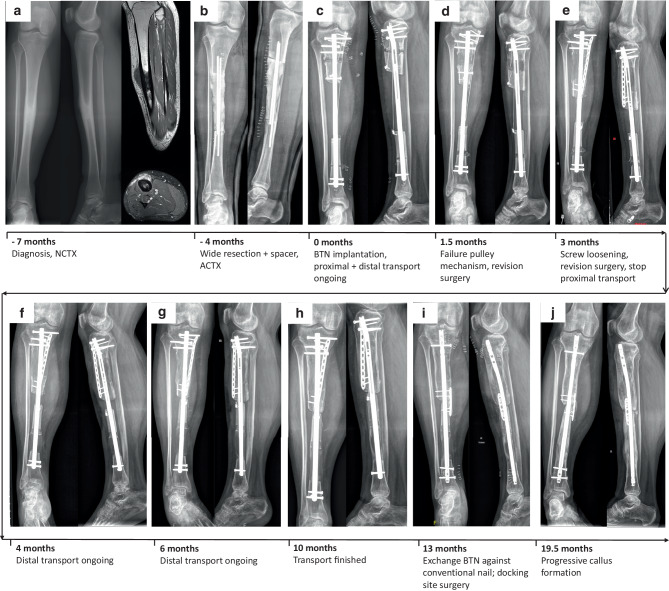
Clinical course of patient 1 (18-year-old male patient with Ewing’s sarcoma of right tibial diaphysis. Images of patient 1 showing (**a**) preoperative x-rays in anterior-posterior (a.p.) and lateral planes together with two magnetic resonance imaging (MRI) scans in sagittal (top) and axial (bottom) of the right tibia. X-ray images in a.p. and lateral planes following wide resection and spacer implantation (**b**), directly after implantation of the bone transport nail (BTN; **c**), at 1.5 months following BTN implantation when the pulley mechanism failed (**d**), at 3 months when another revision surgery due to screw loosening became necessary (**e**), at 4 (**f**) and 6 months (**g**) with bone transport ongoing, at 10 months with finished bone transport (**h**), at 13 months when the BTN was exchanged against a conventional nail (**i**), and at last follow-up (19.5 months) when progressive callus formation was visible, but no complete bony consolidation had taken place (**j**). (*ACTX* adjuvant chemotherapy,* BTN* bone transport nail;* NCTX* neoadjuvant chemotherapy)

An 18-year-old male patient with Ewing’s sarcoma of the right tibial diaphysis underwent wide resection with clear surgical margins following 9 cycles of neoadjuvant CTX. A temporary spacer was implanted in the bony defect measuring 14.1 cm until completion of 5 adjuvant CTX cycles (Fig. 1a, b). Thereafter, a two-level tibial osteotomy was performed and a BTN 11.5/360 mm implanted. To shorten the transport period, a combined proximal and distal bone transport via a pulley mechanism was constructed (Fig. 1c). For this purpose, a wire was fixed to the proximal fragment, rerouted over a screw secured with a mini-plate in the proximal healthy bone, and again fixed at the distal fragment (Fig. 1c). By redirecting the force emerging from transport of the proximal fragment downwards, the distal fragment is pushed upwards. Following surgery, the patient was allowed 40 kg partial weight bearing. The pulley mechanism for retrograde transport of the distal segment failed 1.5 months after surgery, necessitating revision surgery (Fig. 1d). During this procedure, the simultaneous double osteotomy system was abandoned, aiming at enabling completion of proximal bone segment transport via the screw. In a second procedure 1.5 months later due to screw loosening (Fig. 1e), the proximal segment was fixed with a plate and a retrograde bone segment transport reinitiated via renewed osteotomy of the distal segment and a cable-wire pulley mechanism. Bone segment transport was completed after 10.3 months (Fig. 1f–h). Subsequently, the BTN was exchanged with a conventional tibial nail (Expert Tibial Nail, DePuy Synthes, Johnson&Johnson Medical Products GmbH, Vienna, Austria) and the proximal docking site was prepared with curettage and plate fixation due to the lack of complete consolidation (Fig. 1i). Thereafter, the patient was allowed full weight bearing. At latest follow-up 19.5 months after the start of bone transport, complete and partial bony consolidations (confirmed by CT scan) were present at the distal and proximal segments, respectively (Fig. 1j). The patient was fully weight bearing without any pain and had no evidence of disease.

### Patient 2

**Fig. 2:**
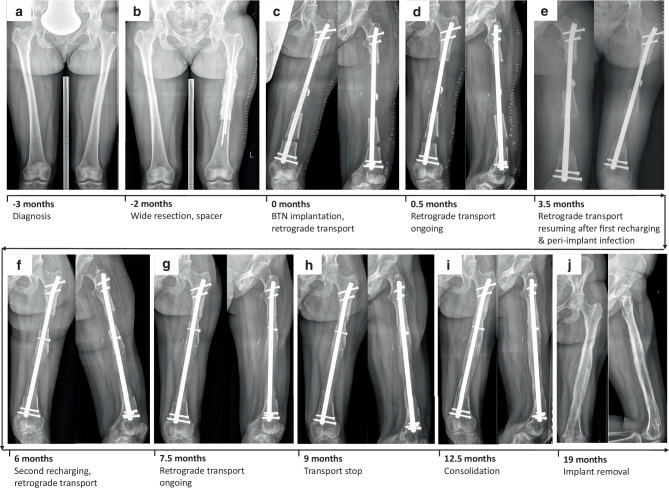
Clinical course of patient 2 (30-year-old female patient with low-grade periosteal osteosarcoma of left femoral diaphysis. X-ray images of both femurs of patient 2 in anterior-posterior (a.p.) plane taken preoperatively (**a**) and after resection and spacer implantation (**b**). X-ray images in a.p. and lateral planes directly following bone transport nail (BTN) implantation (**c**), at 0.5 months (**d**) 3.5 months (**e**), 6 months (**f**) and 7.5 months (**g**) with bone transport ongoing, at 9 months when bone transport stopped (**h**), at 12.5 months when bony consolidation had taken place (**i**), and at latest follow-up 19 months after BTN implantation, when implant removal had taken place and complete bony consolidation was visible (**j**)

Following histological verification of a periosteal osteosarcoma G2 of the left femoral shaft via open biopsy, a 30-year-old female patient underwent surgical resection of the femoral diaphysis (14.4 cm) and temporary implantation of a cement spacer (Fig. 2a, b). After clear margins had been confirmed, reconstructive surgery with distal femoral osteotomy and implantation of a BTN 11.5/360 mm for retrograde transport were performed (Fig. 2c). The patient was allowed 40 kg partial weight bearing until docking (Fig. 2d). Recharging of the BTN was carried out 2.8 months after implantation. The patient developed a peri-implant infection 3 weeks later, necessitating revision surgery with debridement, lavage and antibiotic treatment (initially i.v., later p.o.; Fig. 2e). After the infection had resolved, another recharging was carried out 2 months later (Fig. 2f). At that time point, the patient was allowed partial weight bearing with half body weight. Bone segment transport was completed 9.1 months after BTN implantation and bony consolidation was achieved soon thereafter (Fig. 2g–i), which was confirmed by CT scan. Surgery to exchange the BTN for a conventional femoral nail was carried out 19 months following surgery. Sonication of the explanted nail to rule out the presence of a low-grade implant-associated infection was inconspicuous. Despite a valgus deformity secondary to bone transport of the femur, the patient was free of complaints, fully weight bearing, with no signs of tumor recurrence or infection (Fig. 2j).

### Patient 3

**Fig. 3:**
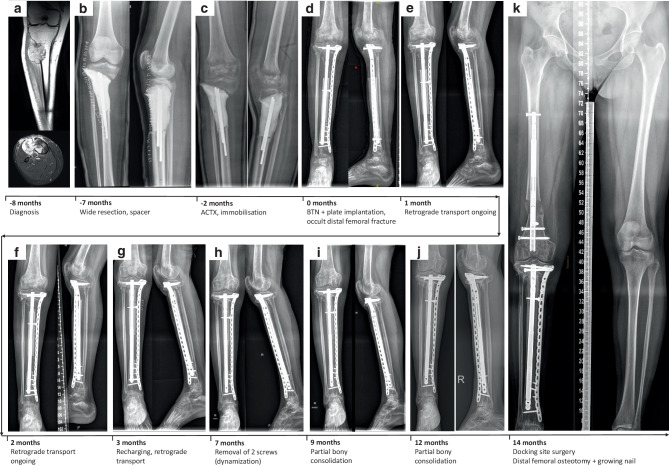
Clinical course of patient 3 (28-year-old female with malignant peripheral nerve sheath tumour (MPNST) of proximal right tibia. Magnetic resonance imaging (MRI) scan in coronal (top) and axial (bottom) plane showing the MPNST at the right proximal tibia (**a**). A.p. and lateral x-ray images following wide resection and spacer implantation (**b**) and during adjuvant chemotherapy with the spacer still in place (**c**). A.p. and lateral x-ray images taken directly following implantation of the bone transport nail (BTN) together with the plate (**d**), at 1 month (**e**), 2 months (**f**) and 3 months (**g**) following BTN implantation, with bone transport ongoing, at 7 months when two screws had just been removed for dynamization (**h**), at 9 months (**i**) and 12 months (**j**) with partial bony consolidation visible, and at last follow-up (14 months) when docking-site surgery had been carried out together with a distal femoral osteotomy and growing nail implantation for varus/flexion deformity correction (**k**). (*ACTX* adjuvant chemotherapy,* BTN* bone transport nail)

A 28-year-old female patient was diagnosed with an epithelioid MPNST originating from the right proximal tibial metaphysis. The diagnosis was ultimately reached in the multidisciplinary team meeting owing to 100% positivity for S100 and SRY-Box Transcription Factor 10 (SOX10) as well as loss of SWI/SNF Related BAF Chromatin Remodeling Complex Subunit B1 (SMARCB1/INI1) expression on immunohistochemistry. Molecular pathological investigations (Archer FUSIONPlex Sarcoma Panel v2; Integrated DNA Technologies, Boulder, CO, USA) revealed no specific genetic fusions. Prior to adjuvant CTX, the patient underwent a wide joint-sparing resection (resulting bone defect 8.5 cm) and implantation of a cement spacer (Fig. 3a, b). Notably, due to the close vicinity of the tumor to the joint, only a small fragment of the tibial epimetaphysis could be preserved. The patient received a full-leg Scotchcast bandage (3M™ Scotchcast, 3M Austria GmbH, 1120 Vienna, Austria) and weight bearing was not allowed (Fig. 3c). Following completion of 6 CTX cycles, the spacer was removed and a BTN 10/300 mm together with a supporting plate implanted (i.e. PABST technique) with retrograde transport of a distal tibial segment (Fig. 3d). Due to prolonged non-weight bearing throughout adjuvant CTX, an occult nondisplaced fracture occurred at the right distal femur (Fig. 3d). The fracture was treated conservatively in a full-leg brace and 15 kg partial weight bearing. Subsequent bone densitometry revealed osteopenia, for which the patient was prescribed vitamin K and calcium after counselling with an endocrinologist. Bone transport was initiated as planned on the 8th postoperative day (Fig. 3e, f). The BTN recharging was performed 3.2 months after initial surgery and the patient was allowed 15 kg partial weight bearing (Fig. 3g). Transport was finished 5.2 months after BTN implantation. To enhance healing, the two distal screws were subsequently removed (Fig. 3k) and 12 months after BTN implantation and 18 months after tumor surgery, partial bony consolidation had taken place (confirmed by CT scan), with progredient callus formation visible at the proximal docking site (Fig. 3i, j). To enhance healing and address the remaining ipsilateral limb length discrepancy as well as varus/flexion deformity of the distal femur, revision surgery with distal femoral osteotomy and implantation of an intramedullary growing nail was carried out 14 months after BTN implantation (Fig. 3k). During the same procedure, autologous cancellous bone grafting at the proximal tibial docking site was carried out. Currently, the distal femoral lengthening procedure is ongoing, the patient is fully weight bearing without complaints and there is no evidence of recurrent disease.

## Discussion

This article reports the preliminary outcomes of three patients with sarcomas of the lower limbs, who underwent defect reconstruction with a BTN. All patients developed one or more complications, either related to the procedure itself or to prolonged non-weight bearing. In all three patients, at least partial bony consolidation had been reached at the latest follow-up. Each case had its own peculiarities and pitfalls that had to be considered during preoperative planning and in the follow-up.

Similar to our series, Copp et al. [13] and Zuckerman [12] described the use of the Precice BTN to reconstruct diaphyseal bone defects in patients with bone sarcomas [12, 13], benign bone lesions [12] and metastases [12]. Other than in our series, bone segment transport was carried out during adjuvant CTX in an adolescent patient with osteosarcoma of the distal femur [12, 13]. Although Bone regenerate formation was observed to some extent during CTX. However, bony regeneration only proceeded sufficiently after CTX had been completed [12]. Comparable findings have been made for bone transport with external fixators, although being generally feasible during cytotoxic CTX, transport time may be prolonged due to slow regenerate formation [11, 14]. At the same time, potential complications developing during bone transport can impair continuation of CTX; therefore we decided not to perform lengthening during systemic treatment (patients 1 and 3). With a cement spacer in place, weight bearing was not allowed during this time. The resulting loss of bone density may have caused the occult intraoperative fracture of the distal femur in patient 3.

In patient 3, additional plating, equivalent to a PABST technique, was carried out due to limited bone stock at the proximal tibia. Other than in the series by Olesen et al., we used a BTN and not a bone lengthening nail for the PABST [3]. One advantage of our method could be the better control of potential axis deviations occurring during bone transport, given that the segment is transported over a rigid nail rather than the nail itself pushing the segment downwards; however, varus/valgus deformities can still occur during bone transport with the BTN (as in ID 2) that can be prevented by using blocking screws prior to BTN insertion [10].

Similar to previous studies, the bone transport time in our series was influenced by the bone defect length, ranging between 5.3 months (defect length 8.5 cm) and 10.3 months (defect length 14.1 cm) [3, 11]. In all three patients, at least partial consolidation was present at the latest follow-up. Notably, all three patients developed at least one complication, either associated with the reconstruction itself (patient 1), repeated surgery (patient 2) or a prolonged period of immobilization (patient 3). The frequency and variability of complications as well as the required bone transport time, highlight the necessity of thorough patient counselling prior to surgery, close monitoring during the perioperative and postoperative period, and strengthen our concept to withhold the lengthening procedure until CTX has been finished. Notably, patients are at risk of developing osteopenia/osteoporosis due to the prolonged immobilization time (potentiated by oncological treatment) [15], wherefore osteoanabolic substances should be considered.

The limitations of this series include the short follow-up period, Therefore our findings should be considered preliminary. Longer observations of our patients will enable assessment of complete consolidation, durability of the transported segment and development of potential complications. Furthermore, none of our patients underwent lengthening during CTX, prolonging the immobilization period before the BTN could be implanted and bone transport initiated; however, as already outlined, complications occurring during lengthening can necessitate interruption of systemic treatment. Related to the magnetic growing mechanism of the Precise® nail itself, it has to be considered that no local MRI scans are possible during active lengthening. After consolidation of the docking site and sufficient callus formation, MRI is again possible with the nail in situ, though. In addition, we only included adult patients with bone sarcomas, while children and adolescents who are predominantly affected by primary malignant bone tumors, have not yet received a BTN for segment transport for oncological indications at our institution. It has to be noted, though that most malignant bone tumors affecting children do not enable joint-sparing surgery given the small anatomy and vicinity to growth plates. To preserve the joint under these circumstances, epiphyseal distraction techniques as the one developed by Canadell may be used [1, 16]. The technique consists of a type I epiphysiolysis using an external fixator prior to definitive surgery, thus enabling resection with clear margins as well as growth plate and joint preservation [1]. Moreover, this technique can be performed during active CTX, serving as an alternative to the BTN in very young individuals.

According to our series, the Precice® BTN can be used for reconstruction of diaphyseal and metaphyseal long bone defects in patients treated for malignant bone tumors. The noninvasive lengthening and reduced foreign material after bone segment transport has been completed, constitute major advantages of this method over endoprosthetic/intercalary replacements. Yet, complications emerging over time require treatment by experienced surgeons familiar with the technique. Thus, patients have to be counselled regarding potential complications and revision surgery as well as the prolonged immobilization and partial weight-bearing period associated with lengthening.

## Data Availability

The datasets generated and/or analyzed during the current study are not publicly available due to data protection policies but are available from the corresponding author on reasonable request.
